# BeHERE’s effective virtual training to build capacity to support people who use drugs in non-substance use disorder settings

**DOI:** 10.1186/s12954-024-00948-5

**Published:** 2024-02-13

**Authors:** Hope Worden Kenefick, Alexis Wing

**Affiliations:** 1 HWK Consulting, LLC, 305 Hemlock Lane, Barrington, NH 03825 USA; 2 Health Resources in Action, 2 Boylston Street, Suite 400, Boston, MA 02116 USA

**Keywords:** Virtual training, Human service settings, People who use drugs, Evaluation

## Abstract

**Background:**

Human service settings not specifically focused on supporting people who use drugs (PWUD), especially those with a substance use disorder (SUD), such as probation and parole services, homeless shelters, and work re-entry and job training programs, offer a unique opportunity to assist this population and prevent overdose deaths. During the COVID-19 pandemic (pandemic), building capacity in such settings for overdose prevention, harm reduction, and to address barriers to treatment, recovery, and support services required that training vendors use a virtual format. Post-pandemic, virtual training remains a cost-effective and convenient alternative to in-person training. The Behavioral Health and Racial Equity (BeHERE) Training Initiative of Health Resources in Action, which offers eight training modules on prevention, recovery, and harm reduction, delivered 224 online trainings between April 2020 and June 2022.

**Methods:**

A mixed methods evaluation based upon the Kirkpatrick Training Evaluation Model was employed, which utilized post-training (*n* = 1272) and follow-up surveys (*n* = 62), and key informant interviews (*n* = 35).

**Results:**

The findings showed BeHERE’s trainings were relevant, engaging, and satisfying to trainees; increased their knowledge, skills, and confidence; and influenced workplace performance. Some participants also indicated that the training influenced the effectiveness of their work with clients and other staff.

**Conclusions:**

The evaluation identified aspects of training that make a virtual format effective at improving the capacity of non-SUD settings to address substance use and support PWUD. Findings offer insights for those interested in delivery of virtual training, as well as training to influence the practice of human service providers across different settings to support PWUD.

**Supplementary Information:**

The online version contains supplementary material available at 10.1186/s12954-024-00948-5.

## Background

Death from drug overdose is arguably one of the most pressing public health crises in the USA today. Since the 1990s, there have been three notable waves of such deaths due to prescription opioids, heroin, and synthetic opioids [1]. Strategies implemented by federal and state governments over the last several decades to address the overdose crisis include funding to expand treatment options, recovery services, naloxone availability and other harm reduction measures, and overdose rescue and prevention education; all of which were disrupted by the COVID-19 pandemic (pandemic) [2, 3]. Despite these measures, by the end of 2021, at the height of the pandemic, the USA reached a grim milestone of over 100,000 drug overdose deaths in a single year [4]. While increased social isolation, financial stressors, housing instability, burnout among harm reduction and health care workers, [5, 6], and toxicity of the drug supply contributed to the pandemic-era surge, so did disruptions in key treatment (e.g., medication for opioid use disorder and detox programs), harm reduction (e.g., syringe service programs), and recovery (e.g., peer support groups) services [7–10]. Workforce shortages and burnout symptoms also affected staff, and thus service availability, in settings not specifically focused on substance use disorders (SUD), but which serve large numbers of people who use drugs (PWUD), including those with a SUD [11, 12]. We distinguish between PWUD and those with a SUD to encompass the full spectrum of substance use from recreational use to a SUD, as there is risk associated with any level of drug use. While there are organizations that operate along the SUD service continuum (e.g., prevention, intervention, harm reduction, treatment, and recovery) to provide support to this specific population, other human service organizations [13] which will be referred to as “non-SUD” settings, are positioned to support people with any level of drug use within their target population. Such non-SUD settings and their needs related to opioid overdose prevention were identified through a needs assessment conducted by the Behavioral Health and Racial Equity (BeHERE) Initiative in 2017 upon being awarded funds from the Massachusetts Department of Public Health. Findings from the 2017 assessment framed the initial audience for training, which included houses of correction and re-entry programs, housing and homeless service programs, transitional assistance programs, children and family services, and disability services. However, as the opioid overdose crisis has evolved, the audience and trainings needs have expanded to include pregnancy and parenting support programs, libraries, and other public venues.

Staff in non-SUD settings are in a unique position to support PWUD to prevent overdose. For example, among populations experiencing homelessness and those that have been incarcerated, there is a greater prevalence of SUD, risk of opioid overdose, and risk of death from overdose [14, 15]. The criminalization and surveillance of drugs and people who use them has contributed to greater arrest and incarceration rates of people with SUD [16]. Thus, it is essential to ensure program staff in non-SUD settings have the skills and knowledge needed to effectively engage and support PWUD. Such preparation must go beyond basic overdose rescue and prevention. Staff must be prepared to address drug-related stigma, provide effective supervisory support to frontline service providers, and mitigate the effects of secondary trauma. Unfortunately, access to SUD-related capacity building services such as training and technical assistance in overdose rescue, prevention, harm reduction, and recovery-related topics was also hindered by the pandemic.

Reported evaluations of SUD-related trainings have demonstrated their efficacy to improve knowledge, confidence, and attitudes related to overdose response and overdose reversal using naloxone [17, 18]. However, these evaluations took place prior to the pandemic, assessed trainings that primarily took place in person, and rarely targeted populations in non-SUD settings. Due to the pandemic, many training programs were forced to transition their activity online, which drove exponential growth in virtual learning opportunities. Despite some challenges associated with virtual training, such as inequities in technology and internet access, post-pandemic it remains a popular, cost-effective, and convenient alternative to in-person training [19]. With overdose deaths continuing to rise in the aftermath of the pandemic and more people turning to the internet for training, it is important to ensure that such training will effectively prepare those in non-SUD settings to engage and support PWUD.

Few studies have assessed the efficacy of virtual SUD-related training in the requisite topics, particularly among those in non-SUD settings. Emerging evidence suggests that online overdose rescue and prevention training can be just as effective as in-person training, and that virtual training can have greater reach and draw increased participation [20–22]. However, these studies narrowly focused on medical students, first responders, or other health care professionals and not on staff in other key non-SUD settings. Findings from an evaluation of the BeHERE Training Initiative help to fill gaps in the existing research. The BeHERE Training Initiative, managed by the Boston-based public health nonprofit agency Health Resources in Action (HRiA), has a mission to build capacity to transform policies and practice in prevention, treatment, harm reduction, and recovery. To that end, BeHERE provides free training and technical assistance to service providers in a range of health and human services organizations across Massachusetts, including non-SUD settings. The Initiative is funded by a State Opioid Response grant from the Substance Abuse and Mental Health Services Administration and the Massachusetts Department of Public Health, Bureau of Substance Addiction Services.

During the pandemic, BeHERE offered eight training modules (see Table 1) on the topics of opioid overdose response, overdose prevention and harm reduction, drug-related stigma, secondary trauma, supervisory best practices, stimulant use, recovery pathways, and the historical context and racist origins of the US War on Drugs. The range of training topics offered by BeHERE is essential to building capacity to prevent overdose and promote harm reduction, particularly among staff in non-SUD settings. Among homeless services workers, supporting effective supervision practices and training has been shown to reduce burnout [12]. Moreover, the impact of stigma or the fear of being stigmatized by providers can lead to poor outcomes, especially among individuals with opioid use disorder, as they may delay seeking treatment or withhold information [23, 24]. However, there is evidence that suggests that training can serve to increase awareness of the ways that stigma among providers can impact care [24].

**Table 1:** BeHERE training module titles and descriptions [25]

Training title	Description	Duration
Opioid overdose rescue training (Part 1)	Teaches about opioids and risk factors for overdose, explores strategies for rescues, and allows participants to practice strategies through scenarios	2 h
Opioid overdose prevention: harm reduction and safety planning with clients (Part 2)	Explores strategies to address overdose risks with a harm reduction approach and includes scenario-based discussions and practice opportunities related to safety, grief, and moving toward behavior change	2 h
Working with people who use stimulants	Teaches participants that, as drug use changes and evolves in Massachusetts and beyond, we need to be prepared to support clients no matter what substance they use. The training teaches the basics of what stimulants are, what they do in the body, and how to support people who use stimulants	3 h
Addressing drug-related stigma and bias	Explores barriers drug-related stigma present to effectively supporting clients who use drugs, identifies the biases in our culture that stigmatize drug use and ostracize those with substance use disorders, and discusses actions to overcome biases and stigma	3 h
Exploring pathways of recovery	Teaches participants that recovery looks different for every person and introduces the various forms of recovery, from medication to 12-step programs to cognitive-based therapies. Participants also explore stigma around recovery and how best to support clients	3 h
Analyzing the US war on drugs and racist drug policies	Explores the historical sources of criminalization and punitive attitudes surrounding drug use in the USA, including in-depth examination of racialized drug policies of the War on Drugs	3 h
Best supervisory practices: working through incidents and crises	Provides supervisors with non-clinical best practices and tools for nurturing and supporting staff who work in substance use, harm reduction, homeless services, and other social service fields, with a particular emphasis on supervisory support following workplace incidents	3 h
Secondary trauma and helping professionals	Covers secondary trauma and cumulative stress with a specific focus on wellness and safety for service providers working in direct care with people who use drugs. Training topics include resilience, PTSD, compassion fatigue, and burnout	3 h

To learn more about the evaluated trainings or BeHERE’s newer trainings, visit: https://behereinitiative.org/trainings/

The BeHERE Training Initiative’s approach to curriculum development and instructional design is informed by the pedagogical work of Paulo Freire, which emphasizes the importance of dialogue, practical knowledge application, and inclusivity in education [26]; Howard Gardner’s theory of multiple intelligences, which suggests that learning materials should be presented in multiple ways to promote students’ understanding of the content [27]; and key principles of adult learning, which emphasize self-direction, respect for learned and lived experience, motivation to solve problems, active involvement in the learning process, and tailoring for participant backgrounds and diversity [28]. These theories and principles are applied in several ways. For example, the lived (past) and living (current) experiences of addiction are central to BeHERE’s trainings. Several of BeHERE’s training facilitators identify as individuals with lived experience of addiction or histories of problematic substance use, and each training begins with the acknowledgement that all participants may be connected to substance use, overdose, or death from overdose in some way. Further, BeHERE promotes active engagement and encourages participants to share their own expertise through large and small group discussions. Participants are also guided through role playing scenarios that may occur in the workplace. Finally, to make training content more accessible to audiences in non-SUD settings, or organizations that do not operate along the SUD services continuum, BeHERE’s trainings are non-clinical. This means that information is not tailored to those with advanced clinical or addiction training (e.g., doctors, psychologists, licensed alcohol and drug counselors, licensed mental health counselors) and thus the trainings provide information and skills useful to both those who provide direct care to clients as well as those who operate at the program development or management level.

BeHERE’s initial training audience was informed by a 2017 needs assessment that identified the need for overdose prevention and related training in non-SUD settings, particularly in organizations not specifically dedicated to the SUD services continuum. At that time (i.e., prior to the pandemic), all trainings were conducted in person, with the idea that in-person training maximizes engagement and best supports the application of the aforementioned theories and principles. In-person trainings were conducted for several state agencies (e.g., the Massachusetts Departments of Transitional Assistance, Mental Health, Children and Families) and many of their affiliated community partners and contracted service providers. To advertise its trainings and to create peer learning opportunities among service providers, BeHERE staff hosted community events throughout Massachusetts which drew human service providers along the SUD services continuum and beyond; participating organizations were added to BeHERE’s marketing distribution list. The onset of the pandemic in March 2020 forced the BeHERE Training Initiative to deliver training virtually while, at the same time, seeking to maintain its approach to instructional design. Early in the pandemic, BeHERE project managers reached out to sites previously involved in in-person trainings to share BeHERE’s new virtual training opportunities. These new virtual training opportunities were also advertised through BeHERE’s regularly updated marketing list. The Bureau of Substance Addiction Services shared information about the virtual training opportunities with state-funded entities, including harm reduction organizations and overdose rescue and naloxone distribution sites. During the pandemic, BeHERE delivered numerous site-specific virtual trainings as well as “open” trainings that enabled individuals from a range of humans services organizations to participate. BeHERE was able to advertise to service providers across the state by getting its trainings posted on multiple training calendars of partner organizations.

In the summer of 2022, BeHERE embarked on a mixed methods evaluation of the eight online trainings delivered between April 2020 and June 2022 to understand if and how it should modify its trainings to make them more satisfying and effective. Findings from the evaluation provide insights about how virtual training delivery for overdose rescue, prevention, and recovery-related topics can build capacity among human service providers, especially those in non-SUD settings, to serve PWUD and individuals with SUD. The evaluation also identified factors that contribute to an engaging virtual training experience, increase knowledge, and influence behavior change among staff in non-SUD settings so they can engage in overdose rescue, prevention, and harm reduction efforts.

## Methods

The evaluation design was based on the Kirkpatrick Training Evaluation Model [29], which suggests that trainings should be evaluated on four levels (see Table 2) and utilized data from participants who completed one or more of BeHERE’s online trainings between April 2020 and June 2022.

**Table 2:** Kirkpatrick training evaluation levels

Level	What is being evaluated?
1	Satisfaction with and engagement in training, and perceived relevance of training to the trainee's job
2	Acquisition of knowledge, skills, and attitudes, as well as confidence about and commitment to use training content
3	Application of what was learned in training when the trainee is back on the job
4	The degree to which targeted outcomes or desired impacts occur as a result of critical on the job behaviors that result from training

The evaluation involved three data collection efforts. Table 3 shows the sample size for each data source as well as the number of trainings completed by participants involved in each data collection effort.

- *Self-administered, online, post-training evaluations* assessing the quality of the training, herein referred to as post-training evaluations (PTE), were completed by trainees at the end of trainings and provided data for evaluating the eight trainings at Kirkpatrick levels 1 and 2. Upon completion of the training, PTEs were administered virtually by emailing links to all training participants with a request to complete the survey in Survey Monkey. In all, 909 unduplicated individuals completed 1272 post-training evaluations, providing evaluation data for 24.9% of the 5308 uses of the training. No incentive was offered to PTE participants. The PTE questions can be found in Additional file 2.
- *Key informant interviews* (KIIs) with 35 individuals who completed one or more trainings provided data for all four Kirkpatrick levels. In all, the key informants completed 69 trainings. Evaluators shared responsibility for participant recruitment. Using contact information of those who completed one or more of the eight trainings during the reporting period, the evaluators reached out via email to solicit KII participation. The evaluators aimed to interview at least five participants from each training to ensure data on all eight trainings were available and that multiple perspectives on each training were gathered. After quickly recognizing that several participants who completed training prior to January 2022 were having difficulty recalling training details, the evaluators shifted to recruitment of those who completed a BeHERE training as of January 2022 or later, approximately six months before the evaluation began. The evaluators shared responsibility for the interviews, with each interviewing roughly half of the participants. The interviewers typed detailed notes during the interviews, including verbatim quotes. At the conclusion of each interview, the interviewers reviewed their notes for gaps and recorded any relevant observations (e.g., changes in tone, issues about which interviewees showed particular passion). All interviews were conducted by telephone using a semi-structured interview tool; each lasted approximately 30 min. Participants were offered an incentive (i.e., a $45 gift card for state employees who are subject to a $49 limit for incentives and $50 gift cards for all others). The key informant interview guide can be found in Additional file 3.
- *Online follow-up survey,* herein referred to as the follow-up survey (FUS), was designed to provide data to evaluate the training on all four Kirkpatrick levels and was completed by 62 individuals, each of whom took one or more trainings for a total of 157 trainings overall. Using contact information for those who completed training during the reporting period, the evaluators reached out via email to request participation in the FUS. The email included a link to a survey administered over a two-week period using Survey Monkey. A reminder email was sent one week after the initial request for participation. Survey participants were eligible to enter a drawing for one of ten $100 gift cards; 27 opted to enter the drawing. The FUS questions can be found in Additional file 4.

**Table 3:** Number of trainees per training, responses available for each data source, and trainings completed

Training	# of training participants*	Post-training evaluations (*n* = 909)	Key informant interviews (*n* = 35)	Follow-up surveys (*n* = 62)
Opioid overdose rescue training (part 1)	1239	189	7	46
Opioid overdose prevention: harm reduction and safety planning with clients and rescue training (part 2)	1086	69	11	24
Opioid overdose rescue and prevention (parts 1 and 2 combined)	**	209	NA	NA
Working with people who use stimulants: best practices	450	137	12	24
Addressing drug-related stigma and bias	618	169	12	18
Exploring pathways to recovery	590	166	8	14
Analyzing the US war on drugs and racist drug policies	438	104	8	13
Best supervisory practices: working through incidents and crises	382	84	6	8
Secondary trauma and helping professionals	505	145	5	10
Total trainings completed by evaluation participants:	5308	1272	69	157

*n for unduplicated trainees across all eight trainings is not available

**n for Parts 1 and 2 are included in counts of training participants above; a subset of trainees evaluated both parts 1 and 2 in a single post-training evaluation submission

The PTE tool was designed and implemented by the BeHERE team prior to involving the external evaluator. Although the evaluator made recommendations for improving post-training data collection (see “Discussion” section), the available data offered utility in describing training participants and assessing level 1 and 2 outcomes. The KII guide and FUS tool were designed by the external evaluator.

As shown in Table 3, between 382 and 1239 participants completed the eight trainings. Because participants often completed more than one training, there is duplication in the total number of training participants, which was 5308 for all eight trainings. The total number of unduplicated trainees across all eight trainings was not available.

The names of more than 290 organizations were listed as places of work by training participants in open-text fields on the PTE. Trainees came from a wide range of organizations across Massachusetts. While many came from programs focused on substance use treatment and/or the provision of recovery supports and services, most came from settings not specifically dedicated to the SUD services continuum, including higher education/academic institutions; state agencies; law enforcement, courts, and parole and probation services; housing/homeless services; public libraries; mental health programs and centers; programs and services for people with special abilities/disabilities; multi-service centers; municipal services; faith-based organizations; and more. A few came from other states or work for the federal government.

**Fig. 1:**
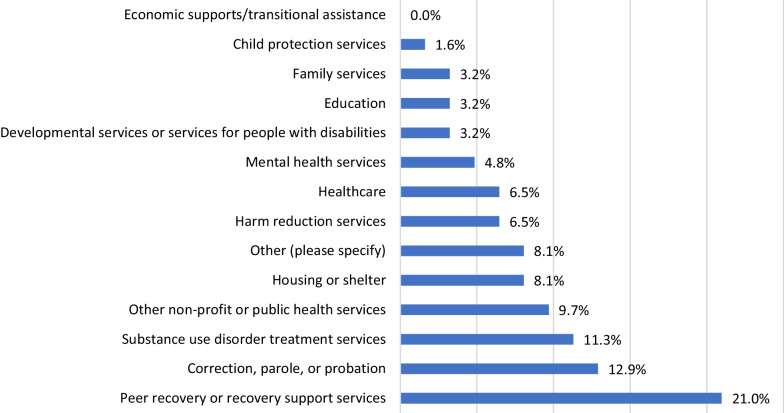
Type of work of FUS respondents (*n* = 62)

Work-related data were also captured on the follow-up survey where respondents were provided with a list of 13 possible work fields and asked to select the one that best describes their type of work. Figure 1 shows that 33.3% work in settings dedicated to SUD treatment or recovery (i.e., 21% in peer recovery programs and 11.3% in SUDs treatment services), the rest work in a range of settings not specifically designed to address SUDs, including corrections, parole, and probation; housing/shelter; healthcare; mental health services; education; and more.

Quantitative data were analyzed using Excel and SPSS to produce relevant frequency distributions. Qualitative data were analyzed for common and divergent themes and illustrative quotes using thematic analysis. The evaluators used a deductive approach with initial coding of their respective interviews influenced by the expected outcomes at each level of the Kirkpatrick Training Evaluation model. For example, at level one, the evaluators identified data related to satisfying and dissatisfying aspects of training, aspects of training that promoted or prevented engagement, and reasons why training was or was not relevant to participants’ work. The evaluators talked regularly throughout the analytic process and, after initial coding of their respective interview notes was completed, exchanged coding documents to identify and resolve discrepancies in how each treated the data. Working together, the evaluators then grouped their agreed upon codes into themes (e.g., about the value and impact of the training, how the training experience could be improved, and other training needs that exist among evaluation participants) and determined which were training-specific versus those that were associated with the BeHERE trainings more broadly. Thereafter, the evaluators determined which quotes to feature in the narrative to illustrate both the perspectives of most interviewees as well as any divergent findings.

## Results

### Level 1: Satisfaction with, engagement in, and relevance of training

In the PTE, participants were asked to rate 10 aspects of the training for quality. As shown in Table 4, 91.5% or more of participants who completed evaluations rated each aspect of the training as good or excellent. The ratings indicate that those who completed PTEs were satisfied with these aspects of the training.

**Table 4:** Percentage of PTE participants who rated aspects of training as good or excellent

Items rated for quality:	Good or excellent (%)
Organization of training (*n* = 1265)	97.9
Usefulness of training to your site (*n* = 1266)	95.0
Trainers/facilitation (*n* = 1264)	98.1
Training materials (*n* = 1261)	96.5
Time allowed for activities (*n* = 1267)	93.4
Technology overview in the introduction (*n* = 1268)	91.6
Overall visual design of course content and materials (*n* = 1267)	93.1
Amount of opportunities for interactive learning (*n* = 1267)	91.9
Use of technology for activities (*n* = 1265)	91.5
Overall online training experience (*n* = 1266)	92.0

On the follow-up survey, participants were asked to indicate their level of agreement (completely disagree to completely agree) with three statements aimed at assessing the trainings for Level 1 effectiveness. Table 5 shows the range for those who completely or somewhat agreed with these statements across all eight trainings. The vast majority of respondents who rated each of the eight trainings expressed agreement that the training(s) they completed were satisfying, engaging, and relevant to their work. For six of the eight trainings, all (100%) of the follow-up survey respondents expressed agreement that the training was satisfying overall and relevant to their work. For five of the eight trainings, 100% of survey respondents offered agreement that the training was engaging.

**Table 5:** Range of percentages who completely or somewhat agreed with Level 1 statements

Statement	Range (%)
Overall, I was satisfied with this training	94.4–100
I found the training to be engaging	91.7–100
The training was relevant to my work	88.9–100

The thematic analysis identified themes across the trainings related to level 1 of the Kirkpatrick model. Below, a sampling of quotes from the various trainings derived from the different sources of qualitative (i.e., key informant interviews, open-ended questions on the post-training evaluation) data illustrates the themes related to level 1 of the Kirkpatrick model.
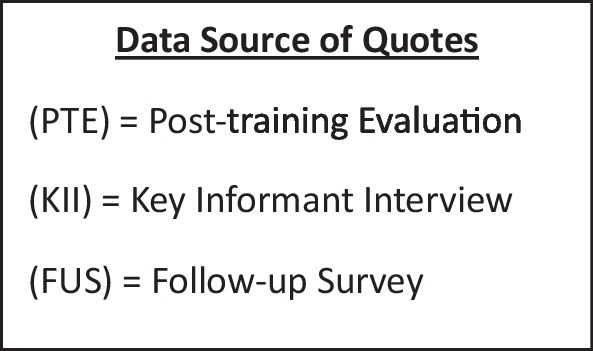


Participants consistently expressed satisfaction with the level of engagement and interaction in the training, noting that they enjoyed learning from the trainers and their colleagues and sharing their own experiences.

*“The sessions were big, over 30 people were in the trainings, and they were done over Zoom and yet they did a phenomenal job of keeping people active and participating.” (KII)*

*“I got to ask questions and there was good dialog between participants and a chance to contribute our own experience and knowledge…” (KII)*

Participants praised the expertise, presentation style, and skills of the trainers, as well as their ability to create a safe and comfortable environment for participants.

*“There was a huge diversity in ideas and opinions, yet the facilitators helped create an environment where everyone felt welcome and comfortable to share.” (PTE)*

*“The facilitators ask thoughtful questions to encourage participants to engage in an honest and authentic way.” (KII)*

In each training, there was specific content and materials (i.e., PowerPoint presentations, tools, and resources) that participants particularly appreciated.

*“There was a chart of how to speak and what words to use and which not to use and how certain words have stigmas. That was probably my favorite part. It was pretty relevant.” (KII)*

*“I liked the materials. I found it very helpful to have links to articles that were cited in the training so I could go back later and read the entire article.” (KII)*

Many participants, across the trainings, were impressed with the technology and how it was used in the trainings, even if they were not enamored of online training or Zoom.

*“[There was] great facilitation and creative use of the Zoom platform to encourage participation.” (PTE)*

*“The annotate function and group brainstorm were effective.” (KII)*

Individuals across all of the trainings found the trainings relevant to their work.

*“The stimulants training was excellent. People coming [to our organization] are not just using opioids; they’re also using meth, crack, and cocaine now.” (KII)*

*“The training I learned today will help me in my day-to-day business. Working in a correctional facility, there are many crises throughout the week affecting both inmates and staff.” (PTE)*

Across the trainings, there were mixed reviews of three things: The use of Zoom versus in-person training; the amount of time allotted for the training content and/or discussion; and the breakout groups (see Table 6).

**Table 6:** Examples of opposing comments about time, use of Zoom, and breakout sessions

Time	*“Have a separate training on motivational interviewing. There wasn’t enough time to practice and learn about it.” (PTE)* *“It was too long and most of us utilize motivational interviewing daily so that was redundant.” (PTE)*
Zoom	*“I thought it was great because, with my job, I wouldn’t be able to go to a lot of the trainings I go to, so Zoom is awesome.” (KII)* *“Zoom is never as satisfying as in-person since it’s more dynamic in person…I’m not crazy about it, but it was effective enough that it kept my interest, and I was engaged.” (KII)*
Breakouts	*“I liked that we were able to go into breakout groups and talk about particular programs and the challenges that we faced.” (KII)* *“[The thing I liked least about the training was] the breakout rooms. Folks keep their cameras off and microphones as well so a group of 4 was only 2.” (PTE)*

While differing opinions existed about time, use of Zoom, and the breakout sessions, the majority of respondents, based upon the PTEs, offered ratings of good or excellent, which suggests that the majority of respondents were satisfied with these aspects of the training: the time allowed for activities (93.4%), overall online training experience (92%), and use of technology for activities (91.5%).

Although opportunities (and suggestions) exist to improve the trainings, the findings indicate that all of the trainings were effective at level 1 of the Kirkpatrick Training Evaluation Model.

### Level 2: Acquired knowledge, skills, attitudes, confidence, and commitment to use training content

The follow-up survey asked respondents to indicate their level of agreement with statements aimed at assessing the level 2 effectiveness of the trainings. For each training, respondents indicated whether “in general” they agreed that the training improved their subject matter knowledge and whether the training taught them new skills or improved their existing skills. Also, for each training, respondents were provided with between four and six statements about knowledge, skills, or abilities specific to the training and were asked to what extent they agreed (completely disagree to completely agree) that those things improved as a result of training. Table 7 shows the range across the eight trainings for those who agreed (somewhat or completely) with the statements. The vast majority of respondents somewhat or completely agreed that the trainings improved their knowledge or skills generally and improved training-specific knowledge, skills, or abilities. For half the trainings, 100% of respondents somewhat or completely agreed that, in general, the training improved their knowledge of the subject matter. For five of the trainings, 100% of respondents somewhat or completely agreed that the training, in general, taught them new skills or improved their existing skills.

**Table 7:** Range of percentages for those who agreed with Level 2 statements about the trainings

Statement	Range (%)
In general, the training improved my knowledge of the subject matter	89.5–100
In general, the training taught me new skills or improved my existing skills	91.7–100
The training improved knowledge, skills, or abilities specific to the training*	85.7–100

*Training-specific findings are available upon request

FUS respondents were also asked training-specific questions about confidence and commitment. For example, survey respondents who completed the Opioid Overdose Rescue training (Part 1) were asked whether they feel more confident about their ability to reverse an opioid overdose *after* training than they felt *before* taking the training. The majority of respondents who completed Part 1 (83.3%) somewhat or completely agreed that the training increased their confidence to reverse an opioid overdose.

Similarly, among those who took Addressing Drug-related Stigma and Bias, 94.4% indicated that they somewhat or completely agreed that they feel even more committed to do what they can to address the impact of bias and stigma related to substance use because of the training. Among those who completed Analyzing the US War on Drugs and Racist Drug Policies, 91.7% somewhat or completely agreed that they feel even more committed to do what they can to address punitive and racist drug policies.

The qualitative data also provided insights into the level 2 effectiveness of the trainings. For some, the trainings offered an introduction to content whereas, for others, the trainings offered a refresher or updated information; in both cases, trainees felt the training helped to increase their knowledge.

*“Taking the Part 1 and 2 training in the first few months of working here was really helpful. Fortunately, I haven’t had to respond to an overdose yet, but I feel prepared to do that.” (KII)*

*“I’ve been doing this for a long time, but services are always evolving and changing, so it’s nice to have a refresher and learn things that I wasn’t aware [of] like holistic and alternative recovery pathways.” (KII)*

Based upon their comments, it seems the trainings have also increased the confidence and commitment of many trainees, especially those for whom the training content was new, to use the information presented in the trainings in their work.

*“…just doing the training made me more confident in identifying secondary trauma.” (KII)*

*“This has made me more confident in asking questions of clients since I’ve had so many clients over the last couple of years that are actively using or in recovery. My commitment has increased for sure.” (KII)*

Across the trainings, participants identified a number of ways in which they intend to use what they learned in their jobs, the most common across the trainings was the intention to share the information learned in training with colleagues and/or clients.

*“I am going to share this knowledge with my colleagues so we can all be better at helping our clients.” (PTE)*

*“I will use the information I learned today in future encounters with patients.” (PTE)*

In addition to sharing knowledge with others, trainees expressed the intention to use what they learned to better support staff and clients (e.g., through motivational interviewing, safety planning, using effective and supportive supervision strategies) and to disrupt stigmatizing language, confronting bias, and advocating for institutional and/or policy change.

*“I will advocate for better access to water, condoms, and lube for our programs, as we use the de-escalation tactics discussed.” (PTE)*

*“I will model better ways of self-care for those in my team.” (PTE)*

*“I will make sure that I speak up more when I hear someone using stigmatizing language.” (PTE)*

The evaluation findings indicate that the trainings were effective at level 2 of the Kirkpatrick model, as the majority of participants indicated that the trainings increased their skills and/or knowledge about the subject matter. Most identified ways they intend to apply what they learned in the training, and several indicated that their confidence and commitment to address the subject matter of the training increased as a result of the training.

### Level 3: Application of what was learned in training when back on the job

For each training, there were between two and four specific actions (e.g., administering Naloxone, using motivational interviewing skills, advocating for non-stigmatizing drug policy in the community) that the trainings were designed to promote. The follow-up survey inquired about 25 actions across all eight trainings (see Table S8 in Additional file 1). Respondents were asked to review actions associated with the training(s) they completed and indicate whether they have performed the actions since taking the training and, if so, whether the training helped prepare them to perform the action or not. Among those who indicated they had completed the actions associated with the training(s) they took, the majority indicated that the training prepared them to perform the actions. For all 25 actions, the majority (over 50%) of those who had performed the actions associated with their trainings indicated that the training prepared them to do so. For 23 of the 25 actions, 75% or more of those who performed the actions since completing training believe the training prepared them to perform those specific actions.

Across the data sources, for each of the eight trainings, respondents offered specific examples of actions they have taken since the training that they explained were influenced by the training.

*“Definitely [I learned and use] de-escalation all the time with patients.” (KII)*

*“I have definitely used what I learned. I now carry Narcan as a result of being trained on how to use it…” (KII)*

*“Recently, I had a person say to me that a person using MAT was not in recovery and that they were just replacing one drug for another. This gave me an opportunity to teach about meeting people where they are and how medication assisted treatment helps to not only prevent overdose but actually sustains recovery.” (FUS)*

*“The Pathways to Recovery [training] also gave me more insight. I’m a 12-stepper so never really looked for other pathways. The training really helped me because I can now help clients to explore their options if the 12-step program doesn’t really work for them.” (KII)*

The evaluation findings indicate that the trainings were effective at level 3 of the Kirkpatrick model among those who had taken training-specific actions since the training. Participants from each of the trainings identified ways in which they applied what they learned in the training in their work.

### Level 4: Targeted outcomes resulting from on-the-job application of what was learned in training

Establishing a relationship between training and desired long-term outcomes is difficult due to the range of confounding variables that affect such outcomes. Six individuals across four trainings (Opioid Overdose Prevention Parts 1 and 2, Best Supervisory Practices, and Working with People who Use Stimulants) described desirable outcomes that occurred since the training with some attributing the outcomes to what they learned in the training.

One key informant, who completed the Best Supervisory Practices training, indicated that the training helped increase efficiency and effectiveness of supervision for recovery coaches. Another experienced the following with a supervisee.

*“…I delegate a lot. I am a nurse and she [the supervisee] is a CHW [Community Health Worker]. She had a different idea of how I should delegate to her. What I was doing was confusing her. That’s going better. Also, [I’m practicing] when to be directive versus supportive.” (KII)*

For the Working with People who Use Stimulants training, a participant indicated that the techniques learned in training helped to de-escalate a situation with a client.

*“A client came in who has a history of using stimulants and was in total uproar and very energetic and jumping around. I was able to identify this behavior as a possible relapse/use of stimulants. I used the de-escalation skills learned from this training to bring the client down a few notches to be able to understand and help the client.” (FUS)*

While three follow-up survey participants who completed the Opioid Overdose Prevention Parts 1 and 2 training had a role in supporting reversal of overdoses, it was not clear whether/how the training influenced the actions taken by these participants. For example:

*“There was an overdose in our congregate shelter setting. Staff were able to administer naloxone and rescue breathing until EMTs were able to arrive. The person was resuscitated when EMTs arrived.” (FUS)*

For the remaining four trainings (i.e., Addressing Drug-related Stigma and Bias, Analyzing the US War on Drugs and Racist Drug Policies, Exploring Pathways to Recovery, and Secondary Trauma and Helping Professionals), no data related to level 4 effectiveness were available.

## Discussion

This evaluation is among the first to assess the delivery of virtual overdose prevention, substance use, and harm reduction training during the pandemic. The value added to the existing literature by this evaluation is the focus specifically on the effectiveness of training staff in both settings along the SUD services continuum as well as non-SUD settings, given their unique roles in supporting PWUD. As such, the evaluation fills gaps in the existing research about how to deliver satisfying and effective virtual education about substance use to staff in non-SUD settings. The evaluation also suggests that BeHERE’s virtual trainings are effective at Levels 1, 2, and 3 of the Kirkpatrick model. However, these findings should be considered in the context of several limitations.

First, while it is possible to report the number of individuals who took each individual training, BeHERE’s registration system did not capture sufficient data to enable the evaluators to identify individuals across all eight trainings. Thus, a count of unduplicated users across the eight evaluated trainings is not available nor is the average and range of trainings taken by the training audience overall; it is only possible to report the number of training instances (i.e., the total number of trainings taken) during the reporting period. Therefore, a comparison of outcomes by high utilizers (e.g., those completing multiple trainings) versus those with low utilization (e.g., a single training) was not possible.

Second, all data were self-reported by respondents. Indeed, these data proved valuable in understanding what participants found satisfying about the training, and how they believe it affected their knowledge, skills, confidence, and attitudes, and workplace performance. However, use of pre- and post-tests would have enabled the evaluators to more definitively assess and attribute changes in knowledge and skills to the training itself.

Third, initial interviews caused the evaluators to re-focus recruitment efforts for the key informant interviews. For some interviewees, more than two years had passed since they had participated in training, which affected their ability to accurately recall details of the training. Thus, the evaluators ultimately focused on recruitment of key informants who took BeHERE trainings since January 2022 (within the six months prior to the start of the evaluation in June 2022). Thus, it is difficult to predict how the training affected those key informants who completed training before January 2022 and whether the training had a lasting impact on their confidence or ability to use training content to support PWUD.

Fourth, the participation rates in the evaluation may have been influenced by factors that could potentially bias the results (e.g., participants’ satisfaction with training, how much they recalled about the training, whether or how much the training was relevant to or useful in their work, the promise of an incentive). Responses to the PTE, which was completed by trainees immediately following training, represent 23.9% of training uses. The key informants who participated in interviews offered reflections on roughly 1.3% of training uses and the follow-up survey participants represent 157 uses of training, about 3% of the total uses of training during the reporting period. The evaluators do not know for sure why some participants opted to provide evaluation data while others did not. However, because the findings overall reflect positively on the BeHERE trainings, it is possible that those whose experiences with training were positive were more likely to provide data for the evaluation, whereas those whose experiences were less positive were not inclined to respond to the evaluators’ requests for participation in data collection. If this were the case, the findings would be biased; however, the data still offer insight into useful aspects of the training, why it was satisfying and relevant, and how it has been useful and influenced the work of a subset of trainees. No incentives were offered related to the PTE, which had the highest response rate, whereas participation was lowest among the KII and FUS, for which incentives were offered. Thus, we believe the use of incentives played a minor role, if at all, in potentially biasing the results of the evaluation. The participation rate in the follow-up survey was lower than the evaluators anticipated, perhaps, in part, due to the time lag between training participation and the evaluation. Greater participation in the follow-up survey may have shed additional light on the impact of the training on participants’ knowledge, skills, and confidence, and provided additional details about whether and how training had influenced performance in the workplace.

Fifth, while data exist to suggest the trainings were effective at level 3, such data were limited given the low response rate among follow-up survey participants and key informants associated with some trainings. Data related to level 4 findings were so limited that the evaluators were unable to ascertain the impact of training on desired outcomes. For example, some of the questions in the follow-up survey did not specifically ask respondents to report outcomes they believe could be attributed, at least in part, to the training. Rather, the questions simply asked about outcomes in general, making it unclear whether participants attribute Level 4 outcomes they identified to the training (versus another factor). Thus, the evaluators could not draw conclusions about the relationship of training to some of the desirable outcomes reported by participants.

The PTE was designed and implemented by the BeHERE team prior to engaging an outside evaluator. While the survey proved useful to the evaluation, it utilized multiple text fields which yielded open-ended responses. Open-ended responses required laborious qualitative analysis and, in some instances, yielded insufficient information to understand the exact meaning of participants’ responses. The evaluator suggested that BeHERE staff use the evaluation findings to identify and develop multiple choice options for the PTE and reduce the number of open-ended text boxes.

Future evaluations of BeHERE (or similar) trainings to build the capacity of staff operating along the SUD services continuum and in non-SUD settings should consider the use of pre and post-tests, seek to recruit larger numbers of respondents, aim to understand the lasting impact of the training on participants and their work, and be designed to detect and describe whether and how the training is effective at influencing outcomes. Additionally, a system for capturing the unduplicated user count across trainings should be developed.

## Conclusions

Despite its limitations, the evaluation of BeHERE’s virtual trainings offers useful findings about the demand for and effective content and delivery of virtual training related to serving PWUD for those in organizations operating along the SUD services continuum and in non-SUD settings.

The evaluation identified several aspects of BeHERE’s virtual training model that participants indicated contributed to its effectiveness, and which may be of use to other training vendors that aim to build capacity among a range of human services providers. While the training aligns with best practices for synchronous online learning described in the existing literature [30], participants specifically pointed to the facilitation, use of technology, and engagement as factors that contributed to their satisfaction.

The evaluation, initially intended to inform BeHERE’s quality improvement efforts, gathered suggestions for ways BeHERE’s trainings could be enhanced. Some suggested that, for example, including content and strategies for working with specific at-risk populations (e.g., people engaging in transactional sex) or historically disadvantaged populations (e.g., Black, Indigenous, and People of Color; non-English speakers; the LGBTQIA + community) would make the training more useful. While the BeHERE staff were encouraged to consider such suggestions, the evaluators also noted the importance of balancing the needs of the few against those of the many for whom such recommendations may not apply. There were also recommendations by some to adjust the time allotted for certain trainings or activities within the trainings. The evaluators suggested that the BeHERE staff may need to distinguish between those for whom the training offered information for the first time (e.g., who feel more time was needed) versus those who are more experienced and took the training as a refresher (e.g., who feel less time was needed). In such cases, the BeHERE staff may want to think about how they position the training (e.g., introductory versus advanced), offering different trainings based on participants’ level of experience, and/or how to engage those with more advanced experience in educating their less experienced colleagues.

With regard to demand, of the participants that completed the follow-up survey (*n* = 62), over half (53%) worked in non-SUD settings, including corrections, parole, and probation; housing/shelter; healthcare; mental health services; education; and more. Likewise, among those who completed the post-training evaluation (*n* = 909), most came from non-SUD settings such as academic institutions; state agencies; law enforcement, courts, and parole and probation services; housing/homeless services, public libraries; health care organizations; mental health programs and centers; programs and services for people living with disabilities; multi-service centers; municipal services; faith-based organizations; and more. Attendance from this diverse group at BeHERE trainings reflects a desire for such training among staff in non-substance use settings.

As noted, BeHERE’s trainings are designed to provide staff with information and skills to support individuals across the spectrum of drug use that they may encounter in their programs. The evaluation findings suggest that, by virtue of their participation, staff in non-SUD settings are better positioned to serve those with an existing SUD, those who may be at risk of developing a SUD (e.g., due to underlying trauma, mental health issues, or other factors), and those who may not be at risk of developing a SUD but who may, nevertheless, be at risk of an overdose due to high-risk drug use.

While over half of participants work in non-SUD settings, 47% of participants work in SUD settings. The evaluation showed that the majority of trainees expressed satisfaction and/or agreement with statements about the training. With so little variation and so few expressing dissatisfaction or disagreement, it seems the training was satisfying, relevant, and beneficial to staff in both SUD-focused and non-SUD settings.

There is clearly interest in and a need for accessible and effective training for those positioned to support PWUD. In 2022, deaths from drug overdose reached 109,680 in a single year [31]. As more unpredictable illicit drugs like xylazine, an animal tranquilizer, hit the market, the risk of opioid-related overdose deaths will increase [32] and further illuminate the need for trained staff in both SUD-focused and non-SUD organizations to address the growing crisis.

### Supplementary Information

**Additional file 1.** Table S8.docx is a Word document that includes Table S8. Assessment of whether training prepared FUS respondents to perform training-related actions completed since training, which is referenced on page 21.

**Additional file 2.** BeHERE Post-Training Evaluation Questions.docx is a Word document that includes questions from the post-training evaluation tool.

**Additional file 3.** BeHERE Follow-Up Survey Questions.docx Word document that includes questions from the follow-up survey tool.

**Additional file 4.** Key Informant Interview Guide.docx is a Word document that includes the key informant interview guide.

## Data Availability

The datasets generated and/or analyzed during this study are not publicly available, but are available upon reasonable request. Requests for data related to the BeHERE Training Evaluation should be forwarded to Alexis Wing at awing@hria.org. All requests will be reviewed by the BeHERE Training Team and a response will be provided in writing, including the requested data or explaining why data may not be available in response to the specific request. Those interested in learning more about the trainings evaluated here or newer trainings produced by BeHERE should visit https://behereinitiative.org/trainings/.

## Abbreviations

BeHERE: Behavioral Health and Racial Equity Initiative
FUS: Follow-up survey
KII: Key informant interview
LGBTQIA+: Lesbian, gay, bisexual, transgender, queer, intersex, asexual, and others
PTE: Post-training evaluation
PWUD: People who use drugs
SOR: State opioid response
SUD: Substance use disorder
