# Supplementary material for: BeHERE’s effective virtual training to build capacity to support people who use drugs in non-substance use disorder settings
Source: Harm Reduct J. 2024 Feb 13;21:38. doi: 10.1186/s12954-024-00948-5 (PMC10863279; doi:10.1186/s12954-024-00948-5)
Supplement: Supplementary file 1 — Additional file 1. Table S8.docx is a Word document that includes Table S8. Assessment of whether training prepared FUS respondents to perform training-related actions completed since training, which is referenced on page 21. [file 12954_2024_948_MOESM1_ESM.docx]

# Appendix A. Table 8. Assessment of whether training prepared FUS respondents to perform training-related actions completed since training

|  | Have done action since training | | Among those performing the action, percent who said… | |
| --- | --- | --- | --- | --- |
|  | (#) | (%) | Training prepared them | Training did not prepare them |
| **Opioid Overdose Rescue Training - Part 1 (n=46)** | | | | |
| I educated someone about opioids and/or overdose risk factors. | 29 | 63.0% | 86.2% | 13.8% |
| I assessed whether a person was overdosing on opioids. | 15 | 32.6% | 80.0% | 20.0% |
| I have administered Naloxone. | 8 | 17.4% | 87.5% | 12.5% |
| I have taken steps to rescue someone who was experiencing opioid overdose. | 10 | 21.7% | 90.0% | 10.0% |
| **Opioid Overdose Prevention: Harm Reduction & Safety Planning with Clients - Part 2 (n=24)** | | | | |
| I have discussed harm reduction strategies with someone I thought might be at risk for opioid overdose. | 14 | 58.3% | 92.9% | 7.1% |
| I used motivational interviewing skills. | 15 | 62.5% | 93.3% | 6.7% |
| I have conducted safety planning with someone I thought might be at risk for opioid overdose. | 12 | 50.0% | 91.7% | 8.3% |
| I have taken steps to keep the program and/or workplace safe. | 13 | 54.2% | 100.0% | 0.0% |
| **Addressing Drug-related Stigma and Bias (n=18)** | | | | |
| I have discussed bias and stigma with someone for whom it is a barrier to services and/or sobriety. | 12 | 66.6% | 83.3% | 16.7% |
| I have recognized and worked to correct an implicit bias that I held related to drug use. | 12 | 66.6% | 75.0% | 25.0% |
| I have interrupted or challenged someone who made stigmatizing comments about drug use or people who use drugs. | 12 | 66.6% | 66.7% | 33.3% |
| I have advocated for non-stigmatizing drug policy in my community. | 12 | 66.6% | 58.3% | 41.7% |
| **Analyzing the U.S. War on Drugs and Racist Drug Policies (n=13)** | | | | |
| I have taken steps to advocate for more just policy related to drug use in my community or workplace. | 4 | 30.8% | 75.0% | 25.0% |
| I have applied systems thinking to an injustice I observed in my community. | 8 | 61.5% | 87.5% | 12.5% |
| **Exploring Pathways to Recovery (n=14)** | | | | |
| I have supported someone seeking recovery. | 11 | 78.6% | 81.8% | 18.2% |
| I have educated someone about the various pathways of recovery available in my community. | 11 | 78.6% | 81.8% | 18.2% |
| I have challenged stigma or bias around one or more pathways of recovery in myself or others. | 11 | 78.6% | 90.9% | 9.1% |
| I have utilized skills of motivational interviewing in a conversation about recovery. | 12 | 78.6% | 91.7% | 8.3% |
| **Working with People Who Use Stimulants (n=24)** | | | | |
| I have worked to de-escalate a tense situation or conflict | 10 | 41.6% | 80.0% | 20.0% |
| I have had a conversation with someone about harm reduction and/or treatment options for stimulant use. | 13 | 54.2% | 84.6% | 15.4% |
| **Best Supervisory Practices: Working through Incidents and Crises (n=8)** | | | | |
| I have incorporated the four leadership styles into my approach to supervising staff. | 7 | 87.5% | 100.0% | 0.0% |
| I have provided support to staff following a crisis. | 5 | 62.8% | 100.0% | 0.0% |
| **Secondary Trauma and Helping Professionals (n=10)** | | | | |
| I have used wellness strategies to help prevent the impact of secondary trauma on me and my work. | 10 | 100% | 90.0% | 10.0% |
| I have advocated or implemented collective care strategies in my workplace. | 6 | 60% | 83.3% | 16.7% |
| I have recognized the impacts of secondary trauma on myself or others and taken steps to address them. | 10 | 100% | 90.0% | 10.0% |
