# Supplementary material for: BeHERE’s effective virtual training to build capacity to support people who use drugs in non-substance use disorder settings
Source: Harm Reduct J. 2024 Feb 13;21:38. doi: 10.1186/s12954-024-00948-5 (PMC10863279; doi:10.1186/s12954-024-00948-5)
Supplement: Supplementary file 2 — Additional file 2. BeHERE Post-Training Evaluation Questions.docx is a Word document that includes questions from the post-training evaluation tool. [file 12954_2024_948_MOESM2_ESM.docx]

Appendix B. BeHERE Post-Training Evaluation Questions

|  |
| --- |

Top of Form

1. Which training did you receive today?

- Addressing Drug-Related Stigma and Bias
- Analyzing the U.S. War on Drugs and Racist Drug Policies
- Best Supervisory Practices: Working through Incidents & Crises
- Challenging Narratives: Understanding Alcohol Use from an Equity Lens
- Exploring Pathways of Recovery
- Motivational Interviewing 101
- Motivational Interviewing 201
- Opioid Overdose Prevention (Part 2)
- Opioid Overdose Rescue (Part 1)
- Opioid Overdose Rescue and Prevention (Parts 1 and 2)
- Promising Policies and Practices for Overdose Prevention, Response, and Postvention: A Workshop
- Secondary Trauma & Helping Professionals
- Supporting People Who Use Drugs: Strategies for Service Providers
- Training of Trainers
- Working with People Who Use Stimulants

2. Site or Organization Name:____________________________________________

3. Role:______________________________________________________________________

4. Age

- Under 18
- 18-24
- 25-34
- 35-44
- 45-54
- 55-64
- 65+

5. Which of the following best represents your racial or ethnic heritage?

- Non-Hispanic White or Euro-American
- Latinx or Hispanic American
- Native American or Alaskan Native
- Black, Afro-Caribbean, or African American
- East Asian or South Asian
- Middle Eastern or Arab American
- Other (please specify)___________________________________________

6. On a scale of 1 (poor) to 5 (excellent), please rate the quality of each of the following aspects of the training. Choose the number that best represents your rating of the quality of each aspect of the training. (insert scale of 1=poor to 5=excellent)

|  |  |  |  |  |  |
| --- | --- | --- | --- | --- | --- |
| - Organization of training |  |  |  |  |  |
| - Usefulness of training (to your site) |  |  |  |  |  |
| - Trainers/Facilitation |  |  |  |  |  |
| - Training materials |  |  |  |  |  |
| - Time allowed for activities |  |  |  |  |  |

7. On a scale of 1 (poor) to 5 (excellent), please rate the quality of each of the following aspects of this virtual training. Choose the number that best represents your rating of the quality of each aspect of the training. (insert scale of 1=poor to 5=excellent)

|  |  |  |  |  |  | N/A |
| --- | --- | --- | --- | --- | --- | --- |
| - Technology overview in the introduction |  |  |  |  |  |  |
| - The overall visual design of the course content and materials |  |  |  |  |  |  |
| - Amount of opportunities for interactive learning |  |  |  |  |  |  |
| - Use of technology for activities |  |  |  |  |  |  |
| - Overall online training experience |  |  |  |  |  |  |

Comments:______________________________________________________

8. What did you like best about this training?_____________________________

9. What did you like least about this training?_____________________________

10. To improve this training, I would:_________________________________________

11. How do you plan to use what you've learned in this training?_________________________________________________________________________

12. Please list any topics related to substance use, overdose prevention, or harm reduction that you would be interested in learning more about and discussing. ______________________________________________________________
