# Supplementary material for: BeHERE’s effective virtual training to build capacity to support people who use drugs in non-substance use disorder settings
Source: Harm Reduct J. 2024 Feb 13;21:38. doi: 10.1186/s12954-024-00948-5 (PMC10863279; doi:10.1186/s12954-024-00948-5)
Supplement: Supplementary file 3 — Additional file 3. BeHERE Follow-Up Survey Questions.docx Word document that includes questions from the follow-up survey tool. [file 12954_2024_948_MOESM3_ESM.docx]

**Appendix C. BeHERE Follow Up Survey Questions**

**Please answer the questions below to help us understand whether and how the training(s) in which you participated have been of value to you, your work, your organization, and to the individuals you serve. Once you have completed the survey, you can enter a drawing to win 1 of 10 $100 gift cards.**

1. **Which category BEST describes the type of work you do? (Please select only one)**

- **Child protection services**
- **Corrections, parole, probation**
- **Developmental services or services for people with disabilities**
- **Economic supports/transitional assistance**
- **Education**
- **Family services**
- **Harm reduction services**
- **Healthcare**
- **Housing or shelter**
- **Mental health/Substance Use Disorder Services**
- **Other non-profit or public health services**
- **Peer recovery or recovery support services**
- **Substance use disorder treatment services**
- **Other (please specify) ______________________________**

1. **Which option below BEST describes your role? (Please select only one.)**

- **Direct service provider**
- **Manager or supervisor**
- **Both a direct service provider AND manager/supervisor**
- **Other (please specify)_______________________________**

1. **In which region of Massachusetts do you work? (Please select only one)**

- **North Shore/North of Boston**
- **Boston**
- **Metro West (i.e., Boston suburbs out to route 495)**
- **South Shore/South of Boston**
- **Cape Cod and/or the Islands**
- **Central MA (i.e., between routes 495 and Route 91)**
- **Western MA (i.e., West of Route 91)**
- **Multiple regions or statewide**
- **Outside of Massachusetts (please specify) ___________________**

**The BeHERE initiative offers a range of trainings to support those working with individuals who use substances, those who are experiencing addiction, and/or individuals who may be on a pathway to recovery. Below, you will be asked whether you participated in any of eight trainings. For each training that you completed, you will be asked to answer a series of questions to help us understand how satisfied you were with the training, whether and how it affected your skills and knowledge, and how the training has influenced your work.**

1. **Did you participate in the Opioid Overdose Rescue Training (Part 1)? This** 2-hour training is designed to teach participants about opioids and risk factors for overdose, explore strategies for rescues, and allow participants to practice strategies through scenarios.

€ yes, I completed this training.

€ no, I did not complete this training.

If no, the participant skips question 5.

If yes, the participant is asked the following questions.

4a. Please use the following scale to indicate the extent to which you agree with the following statements about the **Opioid Overdose Rescue Training** (insert scale 1=*completely agree to 5=completely disagree*)

- Overall, I was satisfied with this training.
- I found the training to be engaging.
- The training was relevant to my work.
- In general, the training improved my knowledge of the subject matter.
- The training improved my knowledge about what opioids are.
- The training improved my knowledge about how to assess for opioid overdose.
- The training improved my knowledge about Naloxone.
- In general, the training taught me new skills or improved my existing skills.
- The training improved my ability to recognize a possible overdose.
- The training improved my skills to assess whether someone is overdosing.
- The training improved my skills to administer Naloxone.
- I feel more confident about my ability to prevent an opioid overdose NOW than I did before the training.
- I feel more prepared to follow steps to respond in the event of an opioid overdose.

4b. We are interested in whether you have applied anything you learned from the **Opioid Overdose Rescue Training**. Please review the list of actions below and indicate whether you applied what you learned in the training to perform any of them.

Insert answer options: (*Yes, I have done this since the training and the training helped prepare me to do it; Yes, I have done this since the training, BUT I don’t feel the training helped prepare me for it; and No, I have NOT been in a situation in which this was needed since the training.)*

- I educated someone about opioids and/or overdose risk factors
- I assessed whether a person was overdosing on opioids.
- I have administered Naloxone.
- I have taken steps to rescue someone who was experiencing an opioid overdose.

4c. In your own words, please describe one way in which the **Opioid Overdose Rescue Training** has contributed to your ability to prevent opioid overdose. ___________________________

4d. If you have been involved in reversing an opioid overdose since the training, please briefly describe the experience (without sharing any confidential or identifying information). ________________________

4e. If there are ways in which the **Opioid Overdose Rescue Training** could be improved, please describe here. ____________________________

**5. Did you participate in Opioid Overdose Prevention:**  **Harm Reduction & Safety Planning with Clients (Part 2? This** 2-hour training is designed to help participants explore strategies to address the risks of overdose with a harm reduction approach.  The training includes opportunities for scenario-based discussions and practice opportunities related to safety, grief, and moving toward behavior change.

€ yes, I completed this training

€ no, I did not complete this training

If no, the participant skips to question 6.

If yes, the participant is asked the following questions.

5a. Please use the following scale to indicate the extent to which you agree with the following statements about the **Opioid Overdose Prevention: Harm Reduction & Safety Planning with Clients** training. (insert scale1=*completely agree to 5=completely disagree*)

- Overall, I was satisfied with this training.
- I found the training to be engaging.
- The training was relevant to my work.
- In general, the training improved my knowledge about the subject matter.
- The training improved my knowledge about harm reduction.
- The training improved my knowledge about the Stages of Change Framework.
- The training improved my knowledge about workplace safety.
- In general, the training taught me new skills or improved my existing skills.
- The training improved my skills related to safety planning.
- The training improved my skills related to motivational interviewing.
- I feel more confident about my ability to prevent an opioid overdose NOW than I did before the training.
- I feel even more committed to do what I can to prevent opioid overdoses because of the training.

5b. We are interested in whether you have applied anything you learned from the **Opioid Overdose Prevention: Harm Reduction & Safety Planning with Clients** training. Please review the list of actions below and indicate whether you applied what you learned in the training to perform any of them.

Insert answer options: (*Yes, I have done this since the training and the training helped prepare me to do it; Yes, I have done this since the training, BUT I don’t feel the training helped prepare me for it; and No, I have NOT been in a situation in which this was needed since the training).*

- I have discussed harm reduction strategies with someone I thought might be at-risk for opioid overdose.
- I used motivational interviewing skills.
- I have conducted safety planning with someone I thought might be at risk for opioid overdose.
- I have taken steps to keep the program/workplace safe.

5c. If you believe you have been involved in preventing an opioid overdose by discussing harm reduction strategies, using motivational interviewing skills, and/or by conducting safety planning, please describe a situation (without sharing any confidential or identifying information). ______________________________________

5d. If, since the **Opioid Overdose Prevention: Harm Reduction & Safety Planning with Clients** training, you have taken steps to keep your program and/or workplace safe, please describe what you have done. _________________________

5e. If there are ways in which the **Opioid Overdose Prevention: Harm Reduction & Safety Planning with Clients** training could be improved, please describe here. ____________________________

**6. Did you participate in Addressing Drug-Related Stigma & Bias?** This 3-hour training is about the barriers drug-related stigma present to effectively supporting clients who use drugs.  The training is designed to help participants identify the biases in our culture that stigmatize drug use and ostracize those with substance use disorders, and discusses actions to overcome biases and stigma.

€ yes, I completed the training.

€ no, I did not complete this training.

If no, the participant skips to question 7.

If yes, the participant is asked the following questions.

6a. Please use the following scale to indicate the extent to which you agree with the following statements about the **Addressing Drug-Related Stigma & Bias** training. (insert scale 1=*completely agree to 5=completely disagree*)

- Overall, I was satisfied with this training.
- I found the training to be engaging.
- The training was relevant to my work.
- In general, the training improved my knowledge about the subject matter.
- The training improved my knowledge about biases in our culture and ways our culture stigmatizes drug use.
- The training improved my knowledge about how stigma affects those who use substances, have a substance use disorder, and/or seek help/treatment.
- In general, the training taught me new skills or improved my existing skills.
- The training improved my ability to recognize and confront bias in myself and others.
- I feel even more committed to do what I can to address the impact of bias and stigma related to substance use because of the training.

6b. We are interested in whether you have applied anything you learned from the **Addressing Drug-Related Stigma & Bias** training. Please review the list of actions below and indicate whether you applied what you learned in the training to perform any of them.

Insert answer options: (*Yes, I have done this since the training and the training helped prepare me to do it; Yes, I have done this since the training, BUT I don’t feel the training helped prepare me for it; and No, I have NOT been in a situation in which this was needed since the training.)*

- I have discussed bias and stigma with someone for whom it is a barrier to services and/or sobriety.
- I have recognized and worked to correct an implicit bias that I held related to drug use.
- I have interrupted or challenged someone who made stigmatizing comments about drug use or people who use drugs.
- I have advocated for non-stigmatizing drug policy in my community.

6c. If, since the training, you have interrupted or challenged someone who made stigmatizing comments, please describe the situation (without sharing any confidential or identifying information). ____________________________________

6d. If there are ways in which the **Addressing Drug-Related Stigma & Bias** training could be improved, please describe here. ____________________________

7. Did you participate in **Analyzing the U.S. War on Drugs & Racist Drug Policies**? This 3-hour training explores the historical sources of criminalization and punitive attitudes surrounding drug use in the U.S., including in-depth examination of the racialized drug policies of the War on Drugs.

€ yes, I completed this training.

€ no, I did not complete this training.

If no, the participant skips to the question 8.

If yes, the participant is asked the following questions.

7a. Please use the following scale to indicate the extent to which you agree with the following statements about the **Analyzing the U.S. War on Drugs & Racist Drug Policies** training**.** (insert scale 1=*completely agree to 5=completely disagree*)

- Overall, I was satisfied with this training.
- I found the training to be engaging.
- The training was relevant to my work.
- In general, the training improved my knowledge of the subject matter.
- The training improved my knowledge levels of racism and how they appear in U.S. drug policy.
- The training improved my knowledge about the iceberg framework of systems thinking.
- The training improved my knowledge about punitive attitudes and policy related to drug use developed in the U.S.
- The training improved my understanding of how U.S. drug policy disproportionately impacts people of color.
- In general, the training taught me new skills or improved my existing skills.
- The training improved my ability to advocate for just policy related to drug use in my community.
- I feel even more committed to do what I can to address punitive and racist drug policies.

7b. We are interested in whether you have applied anything you learned from the **Analyzing the U.S. War on Drugs & Racist Drug Policies** training. Please review the list of actions below and indicate whether you applied what you learned in the training to perform any of them.

Insert answer options (*Yes, I have done this since the training and the training helped prepare me to do it; Yes, I have done this since the training, BUT I don’t feel the training helped prepare me for it; and No, I have NOT been in a situation in which this was needed since the training).*

- I have taken steps to advocate for more just policy related to drug use in my community or workplace.
- I have applied systems thinking to an injustice I observed in my community.

7c. If, since the training, you have taken steps to advocate for more just policy related to drug use, please describe the situation (without sharing any confidential or identifying information). ________________________________

7d. If there are ways in which the **Analyzing the U.S. War on Drugs & Racist Drug Policies** training could be improved, please describe here. ____________________________

8. Did you participate in **Exploring Pathways of Recovery?** This 3-hour training recognizes that recovery looks different for every person and introduces the various forms of recovery, from medication to 12-step programs to cognitive-based therapies.  Participants also explore stigma around recovery and how best to support clients.

€ yes, I completed this training.

€ no, I did not complete this training.

If no, the participant skips to the question 9.

If yes, the participant is asked the following questions.

8a. Please use the following scale to indicate the extent to which you agree with the following statements about the **Exploring Pathways of Recovery** training**.** (insert scale 1=*completely agree to 5=completely disagree*)

- Overall, I was satisfied with this training.
- I found the training to be engaging.
- The training was relevant to my work.
- In general, the training improved my knowledge of the subject matter.
- The training improved my knowledge about the definition and guiding principles of recovery.
- The training improved my knowledge about various pathways of recovery from substance use.
- The training improved my knowledge about how stigma can interfere with recovery from substance use.
- In general, the training taught me new skills or improved my existing skills.
- The training improved my ability to support someone seeking recovery whose identity differs from my own.

8b. We are interested in whether you have applied anything you learned from the training. Please review the list of actions below and indicate whether you applied what you learned in the **Exploring Pathways of Recovery** training to perform any of them.

Insert answer options: (*Yes, I have done this since the training and the training helped prepare me to do it; Yes, I have done this since the training, BUT I don’t feel the training helped prepare me for it; and No, I have NOT been in a situation in which this was needed since the training.)*

- I he supported someone seeking recovery.
- I have educated someone about various pathways of recovery available in my community.
- I have challenged stigma or bias around one or more pathways of recovery in myself or others.
- I have utilized skills of motivational interviewing in a conversation about recovery.

8c. If, since the training, you have supported someone seeking recover, please describe the situation (without sharing any confidential or identifying information). ____________________________________

8d. If there are ways in which the **Exploring Pathways of Recovery** training could be improved, please describe here. ____________________________

9. Did you participate in **Working with People who Use Stimulants: Best Practices?**  This 3-hour training recognizes that as drug use changes and evolves, we need to be prepared to support clients no matter what substances they use.  The training offers participants the basics of what stimulants are, what they do in the body, and how we can support people who use stimulants.

€ yes, I completed this training.

€ no, I did not complete this training.

If no, the participant skips to the question 10.

If yes, the participant is asked the following questions.

9a. Please use the following scale to indicate the extent to which you agree with the following statements about the **Working with People who Use Stimulants: Best Practices** training**.** (insert scale 1=*completely agree to 5=completely disagree*)

- Overall, I was satisfied with this training.
- I found the training to be engaging.
- The training was relevant to my work.
- In general, the training improved my knowledge of the subject matter.
- The training improved my knowledge about the history of American stimulant use and policy.
- The training improved my knowledge about different types of stimulants.
- The training improved my knowledge about how stimulants affect the body and mind.
- In general, the training taught me new skills or improved my existing skills.
- The training improved my ability to support someone who is using stimulants.
- The training improved my skills in de-escalation.

9b. We are interested in whether you have applied anything you learned from the **Working with People who Use Stimulants: Best Practices** training. Please review the list of actions below and indicate whether you applied what you learned in the training to perform any of them.

Insert answer options: (*Yes, I have done this since the training and the training helped prepare me to do it; Yes, I have done this since the training, BUT I don’t feel the training helped prepare me for I; and No, I have NOT been in a situation in which this was needed since the training).*

- I have worked to de-escalate a tense situation or conflict.
- I have had a conversation with someone about harm reduction and/or treatment options for stimulant use.

9c. If relevant, please describe how you have used skills learned in the **Working with People who Use Stimulants: Best Practices** training to de-escalate a tense situation or conflict (without sharing any confidential or identifying information). ____________________________________

9d. If there are ways in which the **Working with People who Use Stimulants: Best Practices** training could be improved, please describe here. ____________________________

10. Did you participate in **Best Supervisory Practices:  Working through Incidents & Crises?** This 3-hour non-clinical training is intended to provide supervisors with the best practices and tools for nurturing and supporting staff who work in substance use, harm reduction, homeless services, and other social service fields, with a particular emphasis on supervisory support following workplace incidents.

€ yes, I completed this training.

€ no, I did not complete this training.

If no, the participant skips to the question 11.

If yes, the participant is asked the following questions.

10a. Please use the following scale to indicate the extent to which you agree with the following statements about the **Best Supervisory Practices: Working through Incidents & Crises** training**.** (insert scale 1=*completely agree to 5=completely disagree*)

- Overall, I was satisfied with this training.
- I found the training to be engaging.
- The training was relevant to my work.
- In general, the training improved my knowledge of the subject matter.
- The training improved my knowledge about the roles and responsibilities of a supervisor.
- The training improved my knowledge about the impacts of secondary trauma and practicing self-care.
- The training improved my knowledge of leadership most appropriate for supervising staff in specific contexts.
- In general, the training taught me new skills or improved my existing skills.
- The training improved my ability to support staff following a crisis.

10b. We are interested in whether you have applied anything you learned from the training. Please review the list of actions below and indicate whether you applied what you learned in the **Best Supervisory Practices: Working through Incidents & Crises** training to perform any of them.

Insert answer options: (*Yes, I have done this since the training and the training helped prepare me to do it; Yes, I have done this since the training, BUT I don’t feel the training helped prepare me for it; and No, I have NOT been in a situation in which this was needed since the training).*

- I have incorporated the four leadership styles into my approach to supervising staff.
- I have provided support to staff following a crisis.

10c. If, since the training, you provided support to staff following a crisis, please describe what you have done differently. ____________________________________

10d. If there are ways in which the **Best Supervisory Practices: Working through Incidents & Crises** training could be improved, please describe here. ____________________________

11. Did you participate in **Secondary Trauma & Helping Professionals?** This 3-hour training covers secondary trauma and cumulative stress with a specific focus on wellness and safety for service providers working in direct care with people who use drugs.  Training topics include resilience, PTSD, compassion fatigue, and burnout.

€ yes, I completed this training.

€ no, I did not complete this training.

If no, the participant skips to the question 12.

If yes, the participant is asked the following questions.

11a. Please use the following scale to indicate the extent to which you agree with the following statements about the **Secondary Trauma & Helping Professionals** training**.** (insert scale1= *completely agree to 5=completely disagree*)

- Overall, I was satisfied with this training.
- I found the training to be engaging.
- The training was relevant to my work.
- In general, the training improved my knowledge of the subject matter.
- The training improved my knowledge about collective care and policies that promote a healthy workplace.
- The training improved my knowledge about the impact of secondary trauma and PTSD can have on direct care providers.
- In general, the training taught me new skills or improved my existing skills.
- The training improved my ability to recognize secondary trauma in myself and my co-workers.
- The training improved my ability to support my own wellness or to prevent/address secondary trauma

11b. We are interested in whether you have applied anything you learned from the training. Please review the list of actions below and indicate whether you applied what you learned in the **Secondary Trauma & Helping Professionals** training to perform any of them.

Insert answer options: (*Yes, I have done this since the training and the training helped prepare me to do it; Yes, I have done this since the training, BUT I don’t feel the training helped prepare me for I; and No, I have NOT been in a situation in which this was needed since the training).*

- I have used wellness strategies to help prevent the impact of secondary trauma on me and my work.
- I have advocated or implemented collective care strategies in my workplace.
- I have recognized the impacts of secondary trauma on myself or others and taken steps to address them.

11c. If you have used wellness strategies and/or addressed secondary trauma for yourself or your co-workers since the **Secondary Trauma & Helping Professionals** training, please describe what you have done and how the training influenced what you did. ____________________________________

11d. If there are ways in which the **Secondary Trauma & Helping Professionals** training could be improved, please describe here. ____________________________

12. If there are other types of training that you would find helpful to support you in your work in substance use, harm reduction, homeless services, and other social services, please describe here. _______________________________

For security purposes, please identify the impact below (insert image of a tree)

13. In a single word, please describe the image above.

Thank you for taking this survey. If you would like to enter the drawing for 1 of 10 $100 gift cards, click “ok” below and then click on the link provided.

If selected at random to receive a gift card, you will be able to choose a card from over 150 stores, businesses, and charities.

If you do not wish to be entered into the drawing, click “ok” and then click “done.”

Click here to enter the gift card drawing: giftcarddrawing.
