# Supplementary material for: BeHERE’s effective virtual training to build capacity to support people who use drugs in non-substance use disorder settings
Source: Harm Reduct J. 2024 Feb 13;21:38. doi: 10.1186/s12954-024-00948-5 (PMC10863279; doi:10.1186/s12954-024-00948-5)
Supplement: Supplementary file 4 — Additional file 4. Key Informant Interview Guide.docx is a Word document that includes the key informant interview guide. [file 12954_2024_948_MOESM4_ESM.docx]

**Appendix D: BeHERE Key Informant Interview Guide**

As part of our evaluation of the SAMHSA (Substance Abuse and Mental Health Services Administration) and MA DPH-funded Opioid Overdose Prevention Training Project (now under BeHERE (Behavioral Health and Racial Equity) that Health Resources in Action (HRiA) has been delivering, we are interviewing several people about their perceptions about the training. My records show that:

1. You were a contact person for [INSERT ORGANIZATION NAME] and worked with HRiA so the trainers could deliver [INSERT TYPE OF TRAINING and the DATES] OR
2. You attended an open registration training hosted by HRiA.
3. *(Category A participants only)* Please describe your experience in working with HRiA to arrange to get the training at your organization.
4. I understand that you attended the following training courses with HRiA: [TRAINING NAMES]. Is that correct? Are there any others you remember taking or would like to give feedback on?
5. I would like to understand how you *(and the training participants)* felt about the training.
   1. Was it satisfying overall?
   2. Were there particular aspects of the training that were particularly effective or engaging? If so, which ones?
   3. Was the training relevant for you, your work, and your organization *(and the staff who attended)*?
   4. Were there aspects that could have been improved? If so, what were they?
   5. Is there anything you thought/wished the training would address that it did not? If so, what?
6. How did you feel about the format and delivery of the training(s) you attended?
   1. Was the training an appropriate length at a convenient time of day?
   2. How did you feel about the delivery of virtual training via Zoom?
   3. Were the materials (e.g. slide decks, links, other resources) shared in a satisfactory way? Did you find them helpful? Have you used them/referred them since completing the training?
   4. Was the online content delivered in an engaging and interactive manner?
   5. Do you have any suggestions regarding delivery of future virtual training?
7. How did your *(or your staff’s)* knowledge and skills related to the topics covered change as a result of the training?
   1. Which skills and/or knowledge do you think you *(and the other staff who attended)* gained from the training(s)?
   2. Did the training(s) affect your *(or your staff’s)* confidence related to the topics covered? If so, describe why you think so.
   3. Did the training affect your *(or your staff’s)* commitment to applying the skills gained from the training(s)? If so, describe why you think so.
8. Since the training, how have you *(or others at your organization)* applied what you learned in the training?
   1. Are you aware of any instances in which participants took steps to apply the concrete skills learned in the training (e.g. reversed an overdose, used de-escalation skills, addressed stigmatizing language, practiced motivational interviewing, etc.)? If yes, please describe.
9. Are there additional training needs? If so, please describe.
   1. Are there any specific topics you or your colleagues/staff may be interested in (e.g. youth and harm reduction, addiction/substance use 101)
   2. Are there any specific ways you would like to receive more support (e.g. learning community; networking; technical assistance; coaching; or small group discussions)
10. Is there anything else you would like to share about the training or its impact on participants?
